# Incongruence Between Observers’ and Observed Facial Muscle Activation Reduces Recognition of Emotional Facial Expressions From Video Stimuli

**DOI:** 10.3389/fpsyg.2018.00864

**Published:** 2018-06-06

**Authors:** Tanja S. H. Wingenbach, Mark Brosnan, Monique C. Pfaltz, Michael M. Plichta, Chris Ashwin

**Affiliations:** ^1^Centre for Applied Autism Research, Department of Psychology, University of Bath, Bath, United Kingdom; ^2^Social and Cognitive Neuroscience Laboratory, Centre of Biology and Health Sciences, Mackenzie Presbyterian University, São Paulo, Brazil; ^3^Department of Consultation-Liaison Psychiatry and Psychosomatic Medicine, University Hospital Zurich, Zürich, Switzerland; ^4^Department of Psychiatry, Psychosomatic Medicine, and Psychotherapy, University Hospital Frankfurt, Frankfurt, Germany

**Keywords:** facial emotion recognition, imitation, facial muscle activity, facial EMG, embodiment, videos, dynamic stimuli, facial expressions of emotion

## Abstract

According to embodied cognition accounts, viewing others’ facial emotion can elicit the respective emotion representation in observers which entails simulations of sensory, motor, and contextual experiences. In line with that, published research found viewing others’ facial emotion to elicit automatic matched facial muscle activation, which was further found to facilitate emotion recognition. Perhaps making congruent facial muscle activity explicit produces an even greater recognition advantage. If there is conflicting sensory information, i.e., incongruent facial muscle activity, this might impede recognition. The effects of actively manipulating facial muscle activity on facial emotion recognition from videos were investigated across three experimental conditions: (a) explicit imitation of viewed facial emotional expressions (stimulus-congruent condition), (b) pen-holding with the lips (stimulus-incongruent condition), and (c) passive viewing (control condition). It was hypothesised that (1) experimental condition (a) and (b) result in greater facial muscle activity than (c), (2) experimental condition (a) increases emotion recognition accuracy from others’ faces compared to (c), (3) experimental condition (b) lowers recognition accuracy for expressions with a salient facial feature in the lower, but not the upper face area, compared to (c). Participants (42 males, 42 females) underwent a facial emotion recognition experiment (ADFES-BIV) while electromyography (EMG) was recorded from five facial muscle sites. The experimental conditions’ order was counter-balanced. Pen-holding caused stimulus-incongruent facial muscle activity for expressions with facial feature saliency in the lower face region, which reduced recognition of lower face region emotions. Explicit imitation caused stimulus-congruent facial muscle activity without modulating recognition. Methodological implications are discussed.

## Introduction

Embodied cognition accounts postulate that there are interrelations between bodily actions (e.g., body posture, gestures) and cognitions. When we acquire memory, we store all information of the specific situation (i.e., context, affect, behaviour, etc.) together in a representation of the situation also containing embodiments (Barsalou, 2008). When we experience an aspect of this initial situation, the remaining memory stored in the representation can get activated (Niedenthal, 2007). For example, observing a smile can activate a representation of a situation that contained smiling (e.g., receiving positive news). This representation can include both the accompanying affect (e.g., feeling happy) and its physical components, including physiological responses and facial muscle activations. In support of this idea, observing facial emotional expressions within a laboratory setting has been found to lead to congruency between observers’ and observed facial muscle activation (e.g., Dimberg, 1982; Dimberg and Thunberg, 1998; Dimberg et al., 2000; Hess and Blairy, 2001; Sato and Yoshikawa, 2007; Achaibou et al., 2008; Likowski et al., 2012). This phenomenon of an observer showing implicit facial muscle activation congruent with the muscle activation in the observed emotional face is generally termed ‘facial mimicry’ (for a literature review, see Hess and Fischer, 2014). Such implicit facial mimicry involves unconscious mechanisms (Dimberg et al., 2000), as muscle activations occur automatically and outside of awareness when healthy people perceive emotional facial expressions (Dimberg, 1982). This automatic muscle activation is different to explicit imitation, which involves the deliberate intention to explicitly imitate the expression of another person and awareness about the activity. Based on embodied cognition accounts, the representation of the emotional expression produced in the observer should facilitate facial emotion recognition of the observed expression due to the stimulus-congruency in facial muscle activations.

Support for the idea that stimulus-congruent facial muscle activation facilitates facial emotion recognition comes from a study investigating the effects of actively manipulating facial movements in observers on facial emotion recognition in others. Oberman et al. (2007) compared recognition rates for happiness, disgust, fear, and sadness using an experimental condition where facial mimicry was ‘blocked’ by having participants actively bite on a pen without the lips touching it. Even though the word ‘blocked’ was used, the manipulation actually created constant muscular activity, which served to produce a non-specific steady state of muscle activity interfering with facial mimicry. Oberman et al. (2007) reported reduced recognition of images displaying disgust and happiness from hindering the observer’s facial mimicry by pen-holding, compared to a condition where no facial movement manipulation was performed. Since recognition was impaired for two out of four investigated facial emotion expressions, Oberman et al. (2007) concluded that facial emotion recognition can be selectively impaired when facial mimicry is hindered. The published literature generally supports the link between automatic stimulus-congruent facial muscle activation in observers and facilitated facial emotion recognition in others (Wallbott, 1991; Stel and van Knippenberg, 2008; Neal and Chartrand, 2011; Sato et al., 2013; but see also Blairy et al., 1999; Rives Bogart and Matsumoto, 2010). Many conclude that being able to engage in facial mimicry facilitates recognition based on the congruency between the facial muscle activation in the stimulus and the observer.

Another explanation for diminished recognition accuracy when participants’ facial movements are actively manipulated (e.g., biting on pen) is that active manipulations themselves induce muscle feedback. Considering what is known from the literature on embodied cognition, it should be noted that such facial muscle feedback itself can have an effect on social processes such as facial emotion recognition. When mouth movement is actively manipulated, the activation in the observer’s face does not align with the activation in the observed expression, instead of being stimulus-congruent as during facial mimicry. This conflicting facial muscle activation could be causing interference during the decoding of the expression leading to decreased recognition accuracy. Ponari et al. (2012) investigated the specific effects of facial muscle manipulation location on facial emotion recognition. These authors manipulated participants’ movement of the lower and upper facial muscles and tested the effects on recognition accuracy of individual emotions. In their study, one group of participants bit on a chopstick horizontally without the lips touching it to fix facial movement in the lower face region (and hinder facial mimicry). The other group in the study had two small stickers attached at the inner edge of the eyebrows and were instructed to push the stickers together to fix facial movement in the upper face region. The inducement of steady facial muscle activation in observers (in the lower and upper face region) diminished recognition of the facial emotional expressions with facial feature saliency in the lower and upper face region, respectively. It is thus possible that the effects on facial emotion recognition in the studies by Oberman et al. (2007) and Ponari et al. (2012) were not the result of hindered facial mimicry. Instead, it is possible that the diminished recognition of certain emotional expressions resulted from the stimulus-incongruent muscle feedback induced by the facial muscle manipulations. This effect could result particularly when the facial region of the salient facial feature in the observed emotional expression is being affected by the facial muscle manipulation in the observer and the resulting facial muscle activity in observers is incongruent with the observed facial muscle activation. Further research is needed investigating this stimulus-incongruency interpretation experimentally.

However, if automatic stimulus-congruent facial muscle feedback in observers (i.e., facial mimicry) facilitates facial emotion recognition, it is plausible that more intense and deliberate muscle activation could facilitate decoding of the observed facial expression of emotion even further (e.g., from explicit imitation of observed facial expression). This assumption is supported by the results of a study by Conson et al. (2013). The study showed better facial emotion recognition performance in actors who explicitly imitated the observed facial emotional expressions and used the resulting generated feeling for decoding emotions (in line with embodiment), compared to actors who used contextual information and thus a more knowledge-based approach. Based on this study, it seems that explicit stimulus-congruent facial muscle activation in observers facilitates facial emotion recognition. However, it is unknown whether the two actor groups differed in their facial muscle activity. The usage of facial EMG allows to investigate differences in facial muscle activity between the various experimental conditions that are assumed to affect facial emotion recognition and is thus indicated. Further, participants were actors with specialised training in nonverbal communication, which includes expressing emotions. Thus, further investigation of explicit imitation and its effect on facial emotion recognition in more general population samples is necessary.

A study considering these factors was conducted by Schneider et al. (2013), who investigated facial emotion recognition in a sample of undergraduate students and applied facial movement manipulations while measuring facial EMG. Results showed that explicit imitation of observed facial expression led to earlier accurate recognition in a morphed sequence of emotional expressions compared to a condition where participants were instructed to suppress their own facial expressions. This suppression condition was intended to hinder participants in producing stimulus-congruent facial muscle activation. In the same study, the condition with free facial movement also led to earlier correct emotion recognition than the expression suppression condition. However, explicit imitation did not lead to a significant advantage over the free facial movement condition. These results suggest that suppression of facial muscle activation in observers diminishes facial emotion recognition rather than that explicit imitation enhances recognition. However, the effectiveness of the instruction to suppress any facial muscle is questionable. Indeed, the EMG results showed no difference in facial muscle activation during the expression suppression condition compared to the free facial movement condition. It is possible that the suppression instruction had a recognition-impairing effect due to other mechanisms like cognitive load. Thus, it might be better to actively manipulate facial muscles to being stimulus-incongruent. With the results on explicit imitation from Schneider et al. (2013) being in contrast to reports by Conson et al. (2013), it still remains to be answered whether explicit stimulus-congruent facial muscle activation in observers facilitates facial emotion recognition or a lack of stimulus-congruency diminishes facial emotion recognition.

Published research has included either an explicit imitation condition or a condition where participants held a pen in their mouth, alongside a condition without any facial movement manipulation. Much of the previous research testing the effects of facial muscle manipulations on emotion recognition ability has used either static images or morphed image sequences, which are limited in ecological validity compared to other types of stimuli. Many previous studies have only used a limited number of basic emotion categories, along only two or three muscle sites in the face to measure muscle activity, which limits the measures about emotion processing and activity in the face. The present study is the first report the authors are aware of to include all three experimental conditions in one experiment to assess how facial emotion recognition is affected by explicit facial muscle activation: (1) an Explicit Imitation condition where participants were told to exactly imitate the expressions they saw while they viewed video stimuli of others displaying various emotional expressions, (2) a Pen-Holding condition where participants held a pen tightly with the lips of their mouth while they watched the videos, and (3) a Passive Viewing control condition where participants just passively viewed the videos. The present study also increased the number of emotion categories included (i.e., anger, disgust, fear, sadness, surprise, happiness, embarrassment, contempt, pride) and measured EMG from five different muscle sites (corrugator supercilii, zygomaticus major, levator labii, depressor anguli oris, and lateral frontalis).

The aim of the present study was to induce explicit facial muscle activation and to investigate the effects of actively manipulating facial muscle activity to being stimulus-congruent and stimulus-incongruent on subsequent facial emotion recognition accuracy based on more ecologically valid stimuli. There were three hypotheses: (1) Enhanced facial muscle activity throughout the face was expected to result from the Explicit Imitation condition, and in the muscles of the lower face region from the Pen-Holding condition, compared to the Passive Viewing control condition. (2) It was hypothesised that enhanced congruency of facial muscle activity between the stimuli and observers (Explicit Imitation condition) would facilitate recognition of emotion compared to the Passive Viewing control condition. (3) It was further hypothesised that the Pen-Holding condition would induce stimulus-incongruent facial muscle activity in observers’ mouth region, resulting in poorer recognition of facial emotional expressions with salient facial features in the lower face region compared to the Passive Viewing control condition.

## Materials and Methods

### Participants

A total of 86 university students (43M/43F; Mean age = 19.6, *SD* = 3.6) were recruited through Campus advertising at the University of Bath and represented both Humanities and Science Departments (54 from Humanities and 32 from Sciences). Technical equipment failure resulted in the loss of data for two participants, resulting in a final sample of 84 participants (41M/43F; Mean age = 19.6, *SD* = 3.6). Based on a power analyses using G^∗^Power (Faul et al., 2007) for the planned analyses to test the main hypotheses (i.e., two-tailed paired samples *t*-tests), a sample size of 84 retrieves 0.78 power with an alpha level of 5% and a small effect size of *dz* = 0.3. The majority of participants in the final sample were undergraduate students (*n* = 82), with one participant enrolled in a Master’s Programme and another in a Ph.D. Programme. Two participants reported about a diagnosis of Major Depression and one participant reported about a diagnosis of an Anxiety Disorder. These participants reported to be on medication and not to experience any symptoms of their mental disorders at the time of participation. Thus, these participants were included in the analyses^1^. All participants had normal or corrected-to-normal vision. Ethical approval for the current study was granted by the Psychology Ethics Committee at the University of Bath.

### Material

#### Facial Emotion Videos

The facial emotion recognition experiment included videos from the validated Amsterdam Facial Expression Set – Bath Intensity Variations (ADFES-BIV; Wingenbach et al., 2016), which is an adaptation from the ADFES (van der Schalk et al., 2011). The ADFES-BIV set contains 360 videos: 12 different encoders (7 male, 5 female) each displaying 10 expressions (anger, disgust, fear, sadness, surprise, happiness, contempt, embarrassment, pride, and neutral/blank stare) across 3 expression intensities (low, intermediate, high). The ADFES-BIV includes 10 more videos of one additional female encoder displaying each of the 10 expression categories once for practise trials. An example image for each emotion category can be found in van der Schalk et al. (2011). Each video is 1040 ms in length. For more detail on the ADFES-BIV (see Wingenbach et al., 2016).

#### Electromyography (EMG) Recording

The BIOPAC MP150 System with the Acqknowledge software (Version 4, Biopac Systems, Inc., Goleta, CA, United States) and EMG110C units for each of the five facial muscle sites (corrugator supercilii, zygomaticus major, levator labii, depressor anguli oris, and lateral frontalis) were used for recording of the EMG data. Pairs of shielded surface silver–silver chloride (Ag–AgCl) electrodes (EL254S) filled with conductive gel (saline based Signa Gel) and with a contact area of 4mm diameter were used. The EMG signal was amplified by 2000 and online bandpass filtering of 10 Hz and 500 Hz was applied. Grounding was achieved through the VIN- of the TSD203 (GSR), the data of which is not reported in this paper. The sampling rate was 1000 Hz throughout the experiment.

### Procedure

Participants were tested in a quiet testing laboratory at the University of Bath, and written consent was obtained prior to study participation. Participants were seated approximately 60 cm from the PC monitor. Before EMG electrode attachment, participants’ faces were cleaned with alcohol swabs. The 10 face EMG electrodes were then placed in pairs over the respective muscle sites on the left side of the face, which was done according to the guidelines by Fridlund and Cacioppo (1986). The electrodes of each pair of electrodes were placed in close proximity to each other using double-stick adhesive rings, with the distance being about 1 cm between the electrode centres. EMG was recorded from five different face muscle sites during the whole duration of the testing session. Participants were kept blind about the true purpose of the study of assessing the effect of facial muscle activity on facial emotion recognition. Thus, participants were told that the electrodes would be measuring pulse and sweat response to facial emotional expressions. After all the electrodes were placed on the face, the participants initially watched a short neutral-content video clip lasting 4 min 18 s, in order to facilitate settling into the research session and to reduce any strong feelings they might have had before the testing session (see Wingenbach et al., 2016). Participants then passively watched 90 videos of the ADFES-BIV to assess facial mimicry without a cognitive load; those results will be presented elsewhere (Wingenbach et al., n.d.). Afterwards, participants underwent the facial emotion recognition task, the data of which is presented in this manuscript. The study included all videos from the ADFES-BIV. However, the facial emotion recognition task of the study presented within this manuscript comprised 280 trials including 10 practise videos. Each of the experimental conditions included equal representations for each of the encoders in the videos, the emotion categories, and the expression intensity levels. There were six different versions of the facial emotion recognition experiment, with each one representing a different order of the three experimental conditions. Participants were pseudo-randomly assigned to one of the six conditions, with the sex ratios being balanced across the versions. Counter-balancing the order of the experimental conditions was important, because performance (e.g., accuracy of response) often increases over the course of the experiment (see section “Results”).

There were 90 trials within each of the three different conditions in the facial emotion recognition experiment: (1) Explicit Imitation, (2) Pen-Holding, and (3) Passive Viewing control condition. During the Explicit Imitation condition, participants were instructed to exactly imitate the facial expressions they observed in the videos (including the blank stare in the neutral expression) as soon as they perceived them. For the Pen-Holding condition, participants were told to hold a pen tightly with their lips, with one end of the pen sticking straight out of their mouth, with pressure applied by the lips (but not the teeth). This manipulation aimed to actively induce facial muscle activity, which also would be incongruent with the emotional expressions included in the study with facial feature saliency in the lower part of the face. The experimenter demonstrated to each participant how the pen was to be held in the mouth, and only after the experimenter was satisfied with the pen-holding technique, the experiment was started. The instruction for the Passive Viewing control condition was to simply watch the videos. Each trial started with a blank screen presented for 500 ms, which was followed by a fixation cross for 500 ms appearing in the centre of the screen. Immediately after the disappearance of the cross the stimulus appeared, followed by a blank screen for 500 ms before the answer screen appeared. The methods used for the facial emotion recognition task were the same as those reported in a previous study using the stimulus set and task (Wingenbach et al., 2016). The answer screen contained 10 labels (neutral and the nine emotion categories) included in the experiment distributed evenly across the screen in two columns and alphabetical order. The participant used a mouse-click to choose their answer, and the mouse-click triggered the next trial. The mouse position was variable. Participants were instructed to choose an emotion label promptly. No feedback was provided about the correctness of the answer. (For more detail about the task procedure, see Wingenbach et al., 2016). After completion of the computer-task, participants were debriefed and compensated with either course credit or GBP 7.

### EMG Data Preparation

**FIGURE 1:**
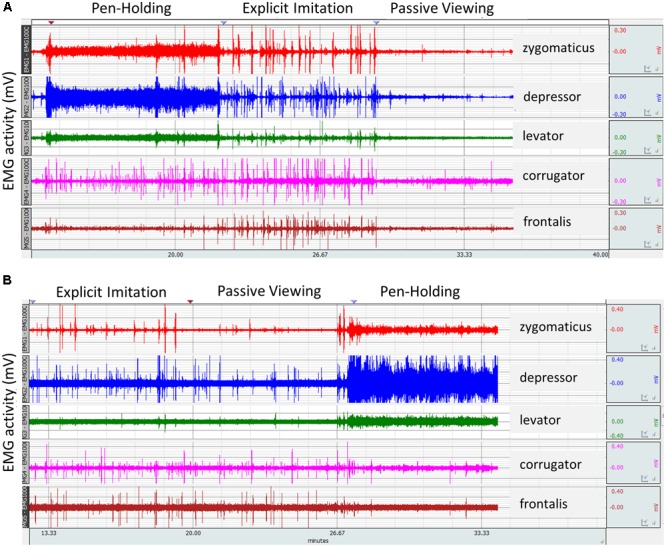
Raw electromyography (EMG) signal from two participants as recorded for the five facial muscles investigated across the three experimental conditions of the study. It was zoomed in at trial level considering stimulus on- and offsets for identification of experimental conditions per participant where task instructions were not fully complied with and thus to exclude from analyses. **(A)** A participant’s EMG activity in compliance with the three experimental conditions. **(B)** Explicit imitation by a participant in the Passive Viewing control condition. Spikes in the EMG signal in the Passive Viewing control condition of similar height as during the Explicit Imitation condition demonstrate explicit imitation instead of passive viewing in the control condition.

Several participants (who did not undergo the Explicit Imitation condition as last condition) verbally self-reported after the testing session that they were unable to stop themselves from imitating the observed facial expressions in subsequent conditions. Thus, the raw EMG data of all participants was visually inspected at trial level to identify participants whose EMG activity pattern suggested explicit imitation in other experimental conditions. Imitative activity on a trial basis during the Passive Viewing control condition was clearly visible in the raw data. **Figure 1** displays the raw data of two selected participants across the whole experiment. Visually comparing the activity in the Passive Viewing control condition from **Figures 1A,B** clearly shows that the participant from **Figure 1B** explicitly imitated in the Passive Viewing control condition. The EMG activity of this participant was, for many trials, as intense in the Passive Viewing control condition as in the Explicit Imitation condition, whereas it should have been similar to the corrugator and frontalis channel during the pen-holding. Eighteen participants were subsequently identified to have shown explicit imitation in conditions other than the Explicit Imitation condition. Looking through the raw EMG data, a further two participants were identified who did not show constant elevated EMG activity in the muscles of the lower part of the face in the Pen-Holding condition, consistent with tightly holding a pen in their mouth (see **Figure 1** as example for the distinctive EMG activation in the first three channels: zygomaticus, depressor, levator). Another participant misunderstood the instructions and did the Explicit Imitation condition twice, so no data on the Passive Viewing control condition exists for this participant. Consequently, the EMG data of these participants for the experimental conditions where the instructions were not fully complied with were excluded from EMG data analyses. The same approach was taken for the accuracy of response data. In addition, there were errors for recording EMG from certain muscles for some participants, which meant the EMG data for some participants was not complete. Again, these participants were still included, but the EMG data of the muscles where problems occurred were excluded from the EMG data analyses. The resulting sample sizes per muscle in each analysis are reported in the respective results section. Participants were not fully excluded from analyses in order to retain enough power for the analyses.

### EMG Data Processing

The Autonomic Nervous System Laboratory 2.6 (ANSLAB; Wilhelm and Peyk, 2005) was used for offline filtering of the EMG data. The EMG signals were 50 Hz notch filtered, 28 Hz high-pass filtered, and the rectified signal was smoothed with a moving average width of 50 ms. A duration of 2.6 s from stimulus onset (excluding the pre-stimulus baseline) was used as the event window, and mean values were calculated and extracted for the event period averaged across all trials with MATLAB (MATLAB 2016b, The MathWorks); this was done for each muscle within each of the three experimental conditions. To assure that the imitation activity was captured within these means, we added 1.5 s to the stimulus offset; a figure demonstrating this necessity based on the activation timings can be found in the Supplementary Figure S1.

## Data Analyses and Results

### Accuracy Changes Across the Experiment Cheque

When participants complete a task consisting of many trials or repeatedly do conditions of a new task, this produces learning effects and the participant’s performance will improve over time. Foroughi et al. (2017) showed that participants shifted from a more effortful approach during a task (which included 48 trials) to a more automatic approach. The faster participants completed a trial and the more trials participants completed, the smaller their pupil dilation became, indicating automatic processing. In the current study, the order of the experimental conditions was counter-balanced for the participants to counter within-task improvements. The accuracy of response data was investigated for the expected within-task improvements over the course of the experiment. This analysis was necessary despite the counter-balancing of the order of the experimental conditions, because the accuracy of response data from the experimental conditions where the instructions were not fully complied with by individual participants (as identified through the EMG data inspection described in section “EMG Data Preparation”) were excluded from further analyses. The elimination of specific conditions for some participants led to unequal numbers of data points per experimental condition. Consequently, the eliminations combined with an increase of accuracy of response over the course of experiments could potentially bias the results. The resulting means for each condition will be inflated for the experimental condition with more data points where this experimental condition was the last condition. Conversely, sample means will be deflated for the experimental condition where more data points factor in from when the experimental condition was undertaken first. Such biases could affect results for any within-subject analyses. It was not foreseeable before data collection that the instruction to explicitly imitate facial emotional expressions would have long-lasting effects on some participants in that they carried over the explicit imitation to subsequent conditions (as described in section “EMG Data Preparation”). Thus, the current study was planned with a within-subject experimental design and respective analyses.

To test for within-task improvements, the individual consecutive trials of the facial emotion recognition task were split into three equal ‘blocks,’ and accuracy of response was calculated for each block in order of their presentation for each participant (i.e., first 90 trials, second 90 trials, third 90 trials). Then, difference scores were calculated between the accuracy of response from the first and second block and between the second and third block. The two resulting difference scores were tested for a significant change using one-sample *t*-tests to test for a significant increase in accuracy of response over the course of the experiment. The alpha-level of 5% was Bonferroni-corrected to account for multiple comparisons; the resulting *p*-values were compared to a *p*-value of 0.025 (*p* = 0.05/2) for significance determination. The within-task improvements analysis was conducted on a sample of *N* = 83 (84 minus one female who did the Explicit Imitation condition twice, as there was no data for this person’s Passive Viewing control condition). Cohen’s *d* is presented as effect size measure. If the results show significant increase in accuracy of response from the first to the second and to the third Block, then this has important implications for the analyses. That is, between-subject analyses with only the first experimental condition each participant completed will be necessary instead of the planned within-subject analyses.

The one-sample *t*-tests showed that there was a significant increase in accuracy of response for participants from the first to the second Block [*M* = 3.69, *SD* = 6.06, *t*(82) = 5.55, *p* < 0.001, Cohen’s *d* = 0.609], and from the second to the third Block [*M* = 1.72, *SD* = 5.15, *t*(82) = 3.05, *p* = 0.003, Cohen’s *d* = 0.335]. Since accuracy scores increased significantly over the course of the experiment, between-subject analyses needed to be conducted to test the hypotheses of the current study. (The results from the within-subject analyses are presented in the Supplementary Figure S2).

### Hypotheses Testing: Facial Muscle Activity Manipulation

To test the effectiveness of the experimental manipulations, the EMG data was statistically examined using generalised linear models for each muscle separately with Experimental Condition included as a factor for each analysis with its three levels (Explicit Imitation, Passive Viewing, and Pen-Holding). Due to the right-skewed nature of the EMG data, gamma distribution and log link function were specified in the conducted analyses. Pairwise comparisons were used to follow up significant main effects of Experimental Condition. Due to the necessary data eliminations described in Section “EMG Data Preparation,” the sample sizes for the EMG data per experimental condition varied. The resulting *n* per comparison are presented with the results.

**FIGURE 2:**
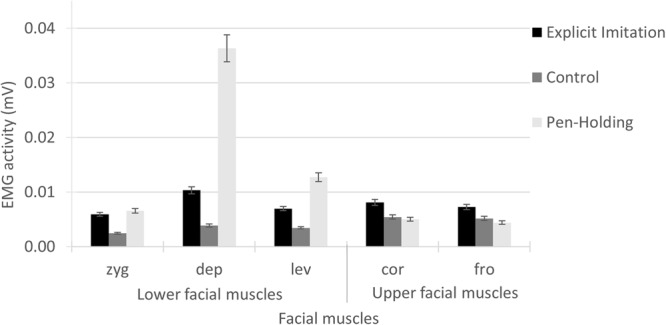
Electromyography activity per facial muscle for each of the experimental conditions. In the Explicit Imitation condition (EI), participants imitated the observed expressions. Participants were holding a pen in the mouth with their lips in the Pen-Holding condition (PH) and passively viewed facial emotional expressions in a Passive Viewing control condition (C). Error bars represent standard errors of the means. zyg, zygomaticus (EI > C < PH); dep, depressor (EI > C < PH); lev, levator (EI > C < PH); cor, corrugator (EI > C = PH); fro, frontalis (EI > C = PH).

Generalised linear model results for the EMG activity in the *zygomaticus* muscle showed a significant main effect of Experimental Condition [Wald χ^2^(2) = 141.79, *p* < 0.001]; see **Figure 2**. Pairwise comparisons showed that the EMG activity in the zygomaticus was significantly higher in the Explicit Imitation condition (*N* = 79, *M* = 0.0059, *SD* = 0.0031) than in the Passive Viewing control condition [*N* = 68, *M* = 0.0025, *SD* = 0.0023, β = -0.88, Wald χ^2^(1) = 1.43, *p* < 0.001], but was not significantly different from the Pen-Holding condition [*N* = 69, *M* = 0.0066, *SD* = 0.0036, β = 0.11, Wald χ^2^(1) = 98.74, *p* < 0.234]. The EMG activity in the zygomaticus during the Pen-Holding condition was significantly higher than during the Passive Viewing control condition (*p* < 0.001).

Generalised linear model results for the EMG activity in the *depressor* muscle showed a significant main effect of Experimental Condition [Wald χ^2^(2) = 538.87, *p* < 0.001]; see **Figure 2**. Pairwise comparisons showed that the EMG activity in the depressor was significantly lower in the Explicit Imitation condition (*N* = 80, *M* = 0.0103, *SD* = 0.0050) than in the Pen-Holding condition [*N* = 70, *M* = 0.0363, *SD* = 0.0243, β = 1.26, Wald χ^2^(1) = 181.33, *p* < 0.001] and significantly higher in the Explicit Imitation condition than in the Passive Viewing control condition [*N* = 69, *M* = 0.0039, *SD* = 0.0028, β = -0.98, Wald χ^2^(1) = 109.86, *p* < 0.001]. The EMG activity in the depressor during the Pen-Holding condition was significantly higher than during the Passive Viewing control condition (*p* < 0.001).

Generalised linear model results for the EMG activity in the *levator* showed a significant main effect of Experimental Condition [Wald χ^2^(2) = 222.39, *p* < 0.001]; see **Figure 2**. Pairwise comparisons showed that the EMG activity in the levator was significantly lower in the Explicit Imitation condition (*N* = 76, *M* = 0.0070, *SD* = 0.0033, *p* < 0.001) than in the Pen-Holding condition [*N* = 67, *M* = 0.0127, *SD* = 0.0083, β = 0.60, Wald χ^2^(1) = 50.44, *p* < 0.001] and significantly higher in the Explicit Imitation condition than in the Passive Viewing control condition [*N* = 66, *M* = 0.0034, *SD* = 0.0019, β = -0.71, Wald χ^2^(1) = 68.62, *p* < 0.001]. The EMG activity in the levator during the Pen-Holding condition was significantly higher than during the Passive Viewing control condition (*p* < 0.001).

Generalised linear model results for the EMG activity in the *corrugator* showed a significant main effect of Experimental Condition [Wald χ^2^(2) = 27.62, *p* < 0.001]; see **Figure 2**. Pairwise comparisons showed that the EMG activity in the corrugator was significantly higher in the Explicit Imitation condition (*N* = 76, *M* = 0.0081, *SD* = 0.0038) than in the Passive Viewing control condition [*N* = 65, *M* = 0.0054, *SD* = 0.0039, β = -0.40, Wald χ^2^(1) = 16.43, *p* < 0.001] and the Pen-Holding condition [*N* = 67, *M* = 0.0050, *SD* = 0.0036, β = -0.48, Wald χ^2^(1) = 23.48, *p* < 0.001]. The EMG activity in the corrugator during the Pen-Holding condition was not significantly different than during the Passive Viewing control condition (*p* = 0.466).

Generalised linear model results for the EMG activity in the *frontalis* showed a significant main effect of Experimental Condition [Wald χ^2^(2) = 26.22, *p* < 0.001]; see **Figure 2**. Pairwise comparisons showed that the EMG activity in the frontalis was significantly higher in the Explicit Imitation condition (*N* = 81, *M* = 0.0072, *SD* = 0.0072) than in the Pen-Holding condition [*N* = 71, *M* = 0.0044, *SD* = 0.0047, β = -0.50, Wald χ^2^(1) = 24.81, *p* < 0.001] and the Passive Viewing control condition [*N* = 70, *M* = 0.0052, *SD* = 0.0044, β = -0.34, Wald χ^2^(1) = 11.35, *p* = 0.001]. The EMG activity in the frontalis during the Pen-Holding condition was not significantly different from the Passive Viewing control condition (*p* = 0.125).

### Hypotheses Testing: Facial Muscle Activity Manipulation and Emotion Recognition Accuracy

Since it was hypothesised that the pen-holding would affect recognition of emotional expressions with facial feature saliency in the lower part of the face but not the upper part of the face, respective variables for the recognition scores were created. The ‘lower face saliency’ variable included accuracy scores for disgust, happiness, embarrassment, contempt, and pride. The ‘upper face saliency’ variable included accuracy scores for anger, fear, sadness, and surprise. This categorisation was based on the location of the facial features that are characteristic for each expression (and the number thereof) in the face stimulus set used; a table listing all facial features per emotion category is printed in van der Schalk et al. (2011). A mean accuracy score was calculated across the emotions included in the lower and upper face saliency variables resulting in a maximum accuracy score of nine (i.e., 100%) each, as there were nine trials per emotion category. Since it was hypothesised that explicit imitation of observed emotional expressions would facilitate recognition of all emotions, the two categories (lower and upper face saliency) were combined to retrieve a recognition score across ‘all emotion categories.’ The maximum possible accuracy score for the latter variable was 18 (i.e., 100%). Whereas analyses were conducted with the accuracy scores, the accuracy scores of the three variables were transformed into percentages in the figures presenting the results to facilitate interpretation.

Only the first experimental condition a participant underwent was included in the between-subject analyses, as the first condition naturally could not have been influenced by former instructions. This between-subject approach decreased the sample size to *n* = 28 for the Passive Viewing control condition (14 male, 14 female) and the Explicit Imitation condition (13 male, 15 female). The sample size was 26 (13 male, 13 female) for the Pen-Holding condition. Three comparisons were conducted using independent samples *t*-tests to test the hypotheses of the current study. The accuracy scores of the variable ‘all emotion categories’ from the Explicit Imitation condition were compared to the accuracy scores from the Passive Viewing control condition to test whether enhanced stimulus-congruent facial muscle activation facilitated recognition. To test whether stimulus-incongruent facial muscle activation impeded recognition, the accuracy scores of the variables ‘lower face saliency’ and ‘upper face saliency’ from the Pen-Holding were compared to the Passive Viewing control condition. The alpha-level of 5% was Bonferroni-corrected to account for multiple comparisons. The resulting *p*-values were compared to a *p*-value of 0.017 (*p* = 0.05/3) for significance determination. Cohen’s *d* is presented as effect size measure.

**FIGURE 3:**
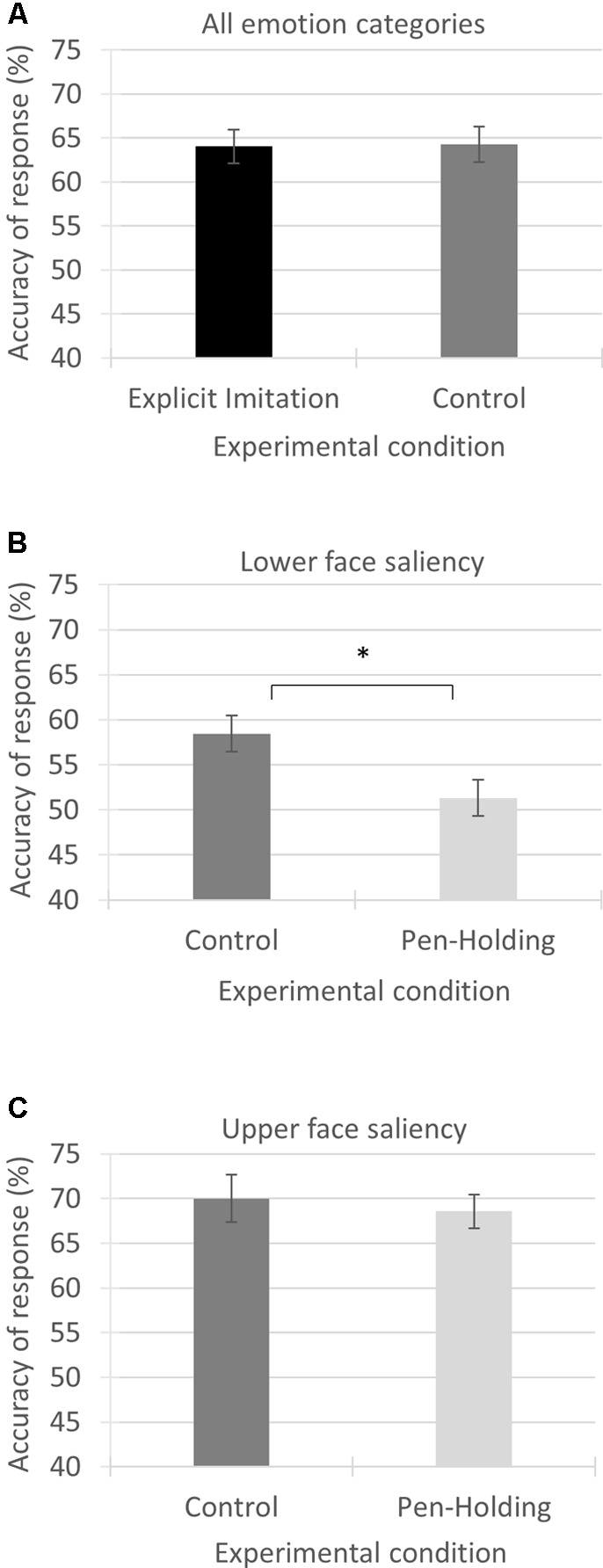
Mean accuracy of response in percentages for the three variables investigated from the experimental conditions as compared. Each panel visualises the results from one of the three conducted comparisons using independent samples *t*-tests. **(A)** Accuracy of response from the Explicit Imitation condition and the Passive Viewing control condition *across all emotion categories*. **(B)** Accuracy of response from the Passive Viewing control condition and the Pen-Holding condition for the *emotion categories with saliency in the lower part of the face*. **(C)** Accuracy of response from the Passive Viewing control condition and the Pen-Holding condition for the *emotion categories with saliency in the upper part of the face*. Error bars represent standard errors of the means. ^∗^*p*-value significant

The independent samples *t*-test comparing accuracy of response across ‘all emotion categories’ included in the task from the Explicit Imitation condition (*M* = 11.53, *SD* = 1.83) to the Passive Viewing control condition (*M* = 11.57, *SD* = 1.91) showed no significant difference between the two experimental conditions [*t*(54) = -0.79, *p* = 0.938, Cohen’s *d* = -0.021]; see **Figure 3A**.

Comparing the accuracy rates of the ‘lower face saliency’ emotion category using independent samples *t*-tests showed that the accuracy rates were significantly higher in the Passive Viewing control condition (*M* = 5.26, *SD* = 0.93) than in the Pen-Holding condition (*M* = 4.62, *SD* = 0.93, *t*(52) = 2.53, *p* = 0.014) with a medium to large effect size (Cohen’s *d* = 0.688); see **Figure 3B**.

Comparing the accuracy rates of the ‘upper face saliency’ emotion category using independent samples *t*-tests showed that the accuracy rates from the Passive Viewing control condition (*M* = 6.30, *SD* = 1.28) were not significantly different from the Pen-Holding condition [*M* = 6.17, *SD* = 0.84, *t*(52) = 0.44, *p* = 0.663, Cohen’s *d* = 0.123]; see **Figure 3C**.

## Discussion

The current study investigated the effects of active facial muscle manipulations in observers on their ability to recognise emotions from others’ faces. Results showed that facial muscle manipulations effectively changed observers’ facial muscle activity. Holding a pen in the mouth increased the activity of facial muscles in the lower face region compared to a control condition with no facial movement manipulation, while explicit imitation of observed facial emotion produced enhanced facial muscle activity across the face compared to the control condition. In line with the facial muscle manipulation, holding a pen in the mouth was found to produce lower accuracy for recognising facial displays of emotion when the most salient facial feature was in the lower face region compared to passively viewing emotional expressions. In contrast, explicitly imitating the emotional expression seen in others did not result in greater recognition of these emotional expressions compared to passive viewing of the videos. The current findings provide support for embodied cognition accounts, but only when the experimental condition involved stimulus-incongruent facial muscle activity while observing emotional expressions in others, and not when the condition involved stimulus-congruent facial muscle activity. The methodological implications for investigations like the current research with a within-subject study design are discussed.

Based on embodied cognition accounts, it was hypothesised that explicit facial muscle activity that is congruent with the observed facial expression would increase recognition rates compared to passive viewing. While explicitly imitating the perceived facial expressions of emotion by others in videos resulted in higher facial muscle activity compared to when they passively viewed the facial expressions, results showed the explicit imitation of others emotions had no facilitating effect on facial emotion recognition. These results are in line with those by Schneider et al. (2013), who similarly reported EMG results showing differences in facial muscle activation between the Explicit Imitation condition and their other two experimental conditions, but no corresponding increase in emotion recognition compared to passive viewing. It was assumed that if automatic subtle stimulus-congruent facial muscle activation facilitated facial emotion recognition (e.g., Oberman et al., 2007), then increasing muscle intensity (i.e., explicit imitation) should increase recognition even more when comparing to a control condition. Though, a study by Hess and Blairy (2001) investigated the intensity of facial mimicry in relation to facial emotion recognition and did not find evidence for a facilitating effect on decoding accuracy due to increased intensity of stimulus-congruent automatic facial muscle activation in observers. Together, these results imply that increased intensity of observers’ stimulus-congruent facial muscle activation does not facilitate recognition. In this case, it is even possible that congruent facial muscle activation in general does not facilitate facial emotion recognition, as reported by Rives Bogart and Matsumoto (2010) based on absent stimulus-congruent facial muscle activity in individuals with face paralysis (i.e., Moebius syndrome) and no different performance at facial emotion recognition compared to non-paralysed controls.

As expected, holding a pen in the mouth caused increased EMG activity in the muscles of the lower face region, especially the depressor muscle. Further in line with the predictions, accuracy scores were significantly lower in the Pen-Holding condition compared to the Passive Viewing control condition when recognising emotional expressions with feature saliency in the lower face region. Effects for mouth movement manipulations on recognition of emotions with saliency in the lower face region are in line with previous studies. For example, disgust and happiness recognition are impaired when mouth movements are manipulated with a pen compared to passive viewing without facial movement manipulation (Oberman et al., 2007; Ponari et al., 2012). For both emotions, the salient facial feature of the corresponding facial expression is situated in the lower face region (mouth and nose, respectively) (Leppänen and Hietanen, 2007; Calvo and Nummenmaa, 2008; Khan et al., 2012). Oberman et al. (2007) interpreted their finding of disgust and happiness recognition being diminished in the Pen-Holding condition compared to the Passive Viewing condition as facial mimicry being a necessary component of facial emotion recognition based on the hindrance of facial mimicry during the Pen-Holding. This explanation does not align with the finding from the current study that stimulus-congruent facial muscle activation did not facilitate recognition.

An alternative interpretation is that facial muscle activations, as achieved through pen-holding, induce facial muscle feedback that is incongruent with the muscle activation underlying the observed facial expression. Since embodiments also include the typical facial expressions of emotions, it was proposed that facial muscle feedback in an observer that is in conflict with the perceived visual information might hamper recognition (Wood et al., 2016). Stimulus-incongruency in facial muscle activation can be determined anatomically. Whereas smiling (through zygomaticus activation for happiness expression) and nose wrinkling (through levator activation for disgust expression) are upward movements, holding a pen in the mouth is an action in the opposite direction, indicating antagonist muscle activation. Importantly, it should be noted that antagonist muscles initiate movement in opposing directions and can thus not be activated simultaneously; this is anatomically impossible (Stennert, 1994). The EMG data from the current study showed that the pen in the mouth induced the greatest muscle activity in the depressor, which indeed is the antagonist muscle to the levator (which itself is a synergist to the zygomaticus). As antagonist muscle, depressor activation produces muscle feedback that is incompatible with smiling/nose wrinkling. The incongruency in facial muscle activation from the pen-holding could have interfered with the embodied representation of the emotions involving facial feature saliency in the lower face region. Observing a facial expression with facial feature saliency in the mouth region (e.g., happiness) would elicit the representation of that emotion, but with a pen in the mouth (i.e., depressor activation), there would be a contradiction in the incoming sensory information. This is because concurrent depressor activation would elicit an association with an emotion whose facial expression involves the depressor. The conflicting muscle activations and the resulting muscle feedback could potentially make recognition of emotional expressions with facial feature saliency in the lower face more difficult. This interpretation aligns with an EEG study that demonstrated that the understanding of facial emotion (i.e., semantic retrieval demand) with facial feature saliency in the lower face region is impaired by active manipulation of muscle activity around the mouth (Davis et al., 2017). Together, these results suggest that recognition is diminished when there is interference between visual and motor information, in line with the wider literature on action-perception matching based on representations (Wohlschläger, 2000; Brass et al., 2001; for a review article see Blakemore and Decety, 2001).

### Limitations, Methodological Considerations, and Future Research

The Explicit Imitation condition and the Pen-Holding condition required additional action from the participants as opposed to the Passive Viewing condition. It could be argued that the results from the current study are based on the additional cognitive load the experimental conditions imposed rather than specific effects of the manipulations. However, Tracy and Robins (2008) demonstrated across two studies that participants are able to accurately recognise emotions, even more complex emotions like pride and embarrassment, under cognitive load. It seems thus unlikely that the findings from the current study are the result of cognitive load. There was a different number of emotional categories included in this study with saliency in the upper part of the face (4) compared to those in the lower face region (5), and this difference could have affected the results.

The current study manipulated the muscles of the lower face region, but not the muscles of the upper face region. Future research should systematically test the effects of stimulus-incongruent muscle activity across the entire face on facial emotion recognition. Researchers have attempted to fix facial muscles in the upper face region by instructing participants to perform certain facial movements (e.g., Ponari et al., 2012). It is likely that such performed facial action (e.g., drawing eyebrows together) is associated with a specific emotional facial expression even if only partially. To overcome this limitation, it could be instructed that participants activate a specific muscle and the effect on recognition of emotional expressions that involve mainly other muscles could be investigated. For example, participants could be asked to smile, frown, wrinkle their nose, etc. each across a set amount of trials displaying varying emotional expressions. Then it could be investigated if stimulus-incongruent facial movements decrease recognition compared to stimulus-congruent expressions. This approach would allow to identify for which muscle interference has the greatest impact on the recognition of specific emotions. These results could have implications for individuals receiving Botox treatments.

Results from the within-subject analyses of the current study showed that the accuracy rates from the Pen-Holding condition were comparable to the Passive Viewing control condition, against the expectation for the emotions with saliency in the lower face region. This finding can, however, be explained by a combination of two occurrences. The first occurrence was the necessary data eliminations, which lead to uneven numbers of participants for the six versions of the experiment. More participants underwent the Passive Viewing control condition first in the experiment sequence than last, while the number of participants per order in the Pen-Holding condition was similar. The second occurrence was the increase in recognition accuracy over the course of the experiment producing higher recognition rates in the last experimental condition a participant underwent. Combining these two occurrences resulted in lower mean accuracy scores for the Passive Viewing control condition, making the mean similar to the mean from the Pen-Holding condition instead of higher. The small albeit non-significant increase in facial emotion recognition when explicitly imitating observed facial expressions compared to the Passive Viewing control condition from the current study can also be explained by the combination of necessary data eliminations and an increase in recognition accuracy over the course of the experiment, as most participants included in the analyses underwent the Explicit Imitation condition last in the experiment. Consequently, theoretical interpretation of the findings from the within-subject analyses of the current study is problematic.

The advantage of a within-subject design is usually that the found effects are the result of the experimental manipulations and not due to potential differences between samples as can be the case in between-subject designs, thereby reducing the error variance. However, the instruction to explicitly imitate the observed facial expressions turned out to have a lasting effect on more than a few participants in the current study. Those participants showed a similar pattern of facial muscle activation in the Pen-Holding condition and Passive Viewing control condition as during the Explicit Imitation condition when the Explicit Imitation condition preceded these conditions. This occurrence indicates that explicit imitation was carried out in the other experimental conditions as well (and led to data loss in the current study). This occurrence is very important to consider for researchers who are intending to conduct research similar to the current study. To avoid data eliminations and potential resulting data confounding effects (see next paragraph), it is advisable to apply a between-subject design. Nonetheless, the instruction to explicitly imitate facial emotional expressions having such a long-lasting effect constitutes an interesting finding in itself. The question why some people automatically keep imitating expressions against the task instructions should gain further attention in future research of this type. Example research questions to address could be: Are these individuals more likely to experience emotion contagion? Do those individuals possess higher empathy?

Further noteworthy is the continuous increase in accuracy of response over the course of the experiment in the current study, independent of the instructions given to participants for the various experimental conditions. The resulting methodological implication is the importance to balance the order of presentation of the experimental conditions when using a within-subject design (as done with the current study) or to apply a between-subject design. The latter option is recommendable when it is likely that unequal amounts of participants will be excluded per order of experimental condition. Nonetheless, that accuracy rates do increase even without the explicit feedback about the correctness of the response is interesting. It indicates some sort of underlying learning processes and it is possible that focussing attention on decoding of facial emotion also outside the laboratory in everyday social interactions might lead to improvements in facial emotion recognition, which could be particularly relevant for clinical populations with impairments in facial emotion recognition.

## Conclusion

Taken together, the current study showed that explicit stimulus-incongruent facial muscle activations in observers hamper recognition compared to passively viewing expressions. It was further demonstrated that explicit stimulus-congruent facial muscle activation does not lead to a facial emotion recognition advantage compared to passively viewing expressions. This latter finding is peculiar since awareness was added to the stimulus-congruent facial muscle activations and the facial muscle activation was explicit (i.e., explicit imitation). Nonetheless, the results from the current study imply that stimulus-congruent facial muscle activations in observers have no facilitating effect on facial emotion recognition and that only stimulus-incongruent facial muscle activations hamper recognition. Given that observing facial emotion might elicit an emotion representation, incongruency between an observed emotion and the facial activity in the observer’s face might disrupt the encoding process due to the embodiment of facial emotional expressions, in line with embodied cognition accounts of emotion.

## Ethics Statement

This study was carried out in accordance with the recommendations of the University of Bath Psychology Ethics Committee with written informed consent from all subjects. All subjects gave written informed consent in accordance with the Declaration of Helsinki. The protocol was approved by the University of Bath Psychology Ethics Committee.

## Author Contributions

TW conceptualised, designed, and carried out the study. MB, CA, and MoP supervised TW. MiP and TW prepared the data for analyses. MoP provided the means for data preparation. TW analysed the data and interpreted the results. TW wrote the manuscript. All authors edited the manuscript.

## Conflict of Interest Statement

The authors declare that the research was conducted in the absence of any commercial or financial relationships that could be construed as a potential conflict of interest.

## Footnotes

1 Analyses on the accuracy data were also conducted excluding these three participants, which had no effect on the outcome of the results.
