# Supplementary material for: Incongruence Between Observers’ and Observed Facial Muscle Activation Reduces Recognition of Emotional Facial Expressions From Video Stimuli
Source: Front Psychol. 2018 Jun 6;9:864. doi: 10.3389/fpsyg.2018.00864 (PMC5997820; doi:10.3389/fpsyg.2018.00864)
Supplement: Supplementary file 1 [file Data_Sheet_1.DOCX]

**Supplementary Material:**

**Incongruence between observers’ and observed facial muscle activation reduces recognition of emotional facial expressions from video stimuli**

Tanja S. H. Wingenbach^*^, Mark Brosnan, Monique C. Pfaltz, Michael M. Plichta, Chris Ashwin

*Correspondence:

Tanja Wingenbach:

tanja.wingenbach@bath.edu

**1 SUPPLEMENTARY FIGURE**


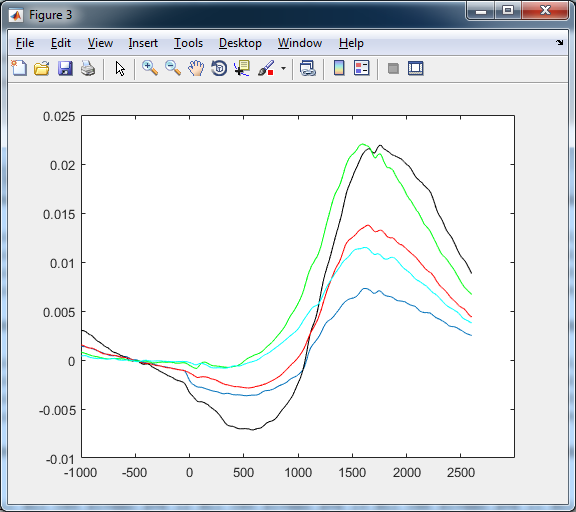


**Figure S1.** The figure shows the mean baseline-corrected EMG signal for the five face muscle sites measured during the Explicit Imitation condition (averaged across all participants and trials). Each EMG channel is displayed in a different colour. The x-axis represents the time course; the y-axis the EMG activation in mv. The 0 on the x-axis represents stimulus-onset. The graph demonstrates that an event window of 2.6 seconds from stimulus-onset allows for capturing of the EMG activity of the explicit imitation period.

**2 SUPPLEMENTARY DATA**

**Within-subject analyses: facial muscle activity manipulation and emotion recognition accuracy**

The study was planned with a within-subject design and thus paired samples *t*-tests conducted to test the hypotheses. Since participants were only excluded for certain experimental conditions, the resulting *n* per comparison varied and are presented in the results. The paired samples *t*-test comparing accuracy of response across ‘all emotion categories’ included in the task from the Explicit Imitation condition (*M* = 12.29, *SD* = 1.98) to the Passive Viewing control condition (*M* = 11.95, *SD* = 1.98) showed no significant difference between the conditions based on the Bonferroni-corrected *p*-value (.017) with a small effect size (*N* = 72 (35 male, 37 female), *t*(71) = 2.03, *p* = .046, Cohen’s *d* = .244); see Figure S2 (A).


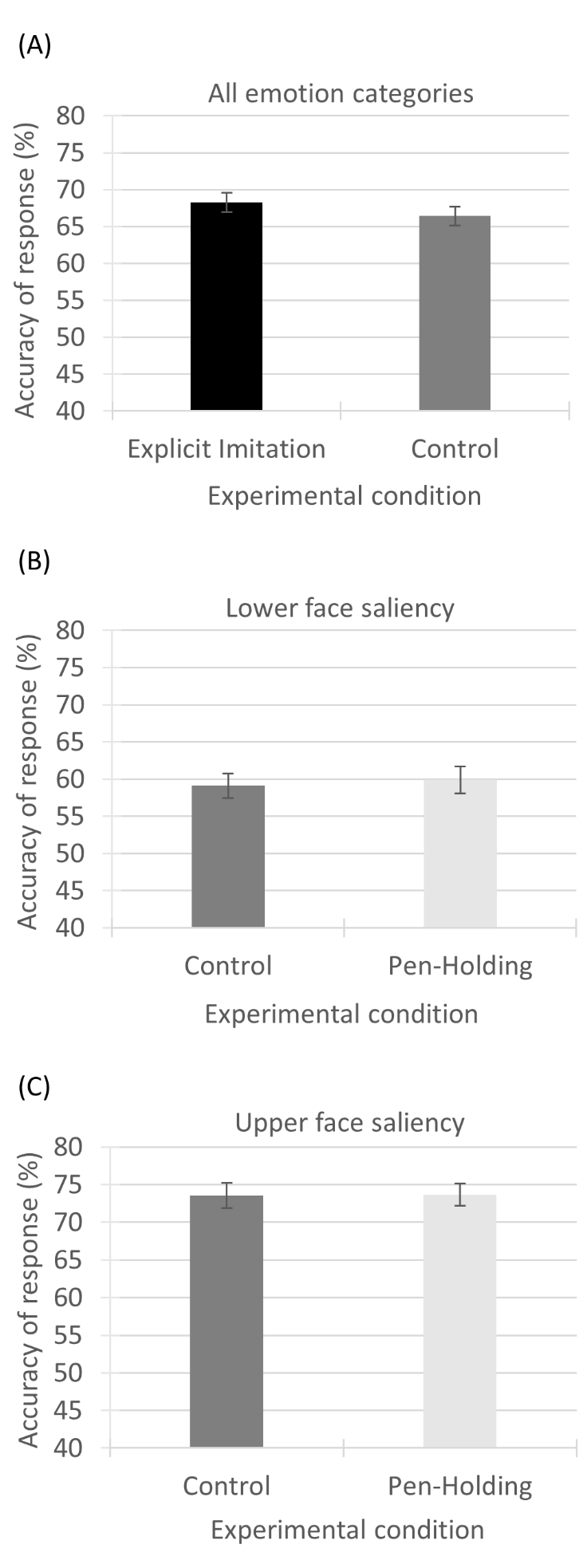


**Figure S2**. Accuracy of response based on the within-subject analyses. **(A)** Accuracy or response from the Explicit Imitation condition and the Passive Viewing control condition across all emotion categories. **(B)** Accuracy of response from the Passive Viewing control condition and the Pen-Holding condition for the emotion categories with saliency in the lower part of the face. **(C)** Accuracy or response from the Passive Viewing control condition and the Pen-Holding condition for the emotion categories with saliency in the upper part of the face. Error bars represent standard errors of the means.

Comparing the accuracy rates of the ‘lower face saliency’ emotion category using paired samples *t*-tests showed that the accuracy rates from the Passive Viewing control condition (*M* = 5.32, *SD* = 1.17) were not significantly different from the accuracy rates of the Pen-Holding condition (*M* = 5.39, *SD* = 1.30, *N* = 63 (31 male, 32 female), *t*(62) = -0.57, *p* = .572, Cohen’s *d* = -.080), see Figure S2 (B).

Comparing the accuracy rates of the ‘upper face saliency’ emotion category using paired samples *t*-tests showed that the accuracy rates from the Passive Viewing control condition (*M* = 6.62, *SD* = 1.18) were not significantly different to the Pen-Holding condition (*M* = 6.63, *SD* = 1.03, *N* = 63 (31 male, 32 female), *t*(62) = -0.10, *p* = .921, Cohen’s *d* = -.010), see Figure S2 (C).
